# Stochastic processes shape microeukaryotic community assembly in a subtropical river across wet and dry seasons

**DOI:** 10.1186/s40168-019-0749-8

**Published:** 2019-10-22

**Authors:** Weidong Chen, Kexin Ren, Alain Isabwe, Huihuang Chen, Min Liu, Jun Yang

**Affiliations:** 1 Aquatic EcoHealth Group, Key Laboratory of Urban Environment and Health, Institute of Urban Environment, Chinese Academy of Sciences, Xiamen, 361021 China; 2 Fujian Key Laboratory of Watershed Ecology, Institute of Urban Environment, Chinese Academy of Sciences, Xiamen 361021, China; 3 State Key Laboratory of Marine Environmental Science, Marine Biodiversity and Global Change Research Center, College of Ocean and Earth Sciences, Xiamen University, Xiamen, 361102 China

**Keywords:** Biogeography, Plankton, Microeukaryotic community, Subtropical river, Environmental factors, Neutral community model, Community assembly

## Abstract

**Background:**

The deep mechanisms (deterministic and/or stochastic processes) underlying community assembly are a central challenge in microbial ecology. However, the relative importance of these processes in shaping riverine microeukaryotic biogeography is still poorly understood. Here, we compared the spatiotemporal and biogeographical patterns of microeukaryotic community using high-throughput sequencing of 18S rRNA gene and multivariate statistical analyses from a subtropical river during wet and dry seasons.

**Results:**

Our results provide the first description of biogeographical patterns of microeukaryotic communities in the Tingjiang River, the largest river in the west of Fujian province, southeastern China. The results showed that microeukaryotes from both wet and dry seasons exhibited contrasting community compositions, which might be owing to planktonic microeukaryotes having seasonal succession patterns. Further, all components of the microeukaryotic communities (including total, dominant, always rare, and conditionally rare taxa) exhibited a significant distance-decay pattern in both seasons, and these communities had a stronger distance-decay relationship during the dry season, especially for the conditionally rare taxa. Although several variables had a significant influence on the microeukaryotic communities, the environmental and spatial factors showed minor roles in shaping the communities. Importantly, these microeukaryotic communities were strongly driven by stochastic processes, with 89.9%, 88.5%, and 89.6% of the community variation explained by neutral community model during wet, dry, and both seasons, respectively. The neutral community model also explained a large fraction of the community variation across different taxonomic groups and levels. Additionally, the microeukaryotic taxa, which were above and below the neutral prediction, were ecologically and taxonomically distinct groups, which might be interactively structured by deterministic and stochastic processes.

**Conclusions:**

This study demonstrated that stochastic processes are sufficient in shaping substantial variation in river microeukaryotic metacommunity across different hydrographic regimes, thereby providing a better understanding of spatiotemporal patterns, processes, and mechanisms of microeukaryotic community in waters.

## Background

Microbial community assembly, the mechanisms shaping the microbial community diversity, and its distribution, functions, succession and biogeography, is a central, but poorly understood, topic in aquatic microbial ecology, especially in lotic ecosystems [1–5]. Niche-based and neutral-based theories constitute two important and complementary mechanisms for understanding microbial community assembly [6–8]. The niche-based processes considers that microbial communities are shaped by the deterministic abiotic (environmental factors such as pH and temperature) and biotic factors (species interactions such as competition and predation) due to different habitat preferences and fitness of microbes [9–11]. On the contrary, the neutral theory asserts that stochastic processes, such as birth, death, immigration, speciation, and limited dispersal, shape the microbial community structure [4, 8, 12, 13], and assumes that microbes exhibit a stochastic balance between the loss and gain of taxa [7, 14]. Although stochastic processes are considered to play important roles in shaping microbial community structure [6–8, 15], the importance of ecological stochasticity in influencing community structure is far less appreciated. The main reasons for such little appreciation are the difficulty in defining stochasticity and the methods used in representing stochasticity [4]. Community diversity and distribution patterns can provide evidence for the processes underlying community assembly [1, 16]. The neutral community model (NCM), proposed by Sloan et al. [7], is particularly useful in quantifying the importance of neutral processes [7, 13].

Most microbial community assembly studies have focused on marine, soil, and lake environments [2, 10, 17–19]; however, microbial plankton investigations of lotic ecosystems (e.g., rivers) are still limited, especially in subtropical region [20]. The river environments are always accompanied with complex biogeochemical and microbial community variations. Indeed, certain development activities, such as agriculture, industry, and urbanization, can affect environmental variables of lotic ecosystems, leading to environmental conditions that are more complex and dynamic compared to lentic ecosystem (e.g., lakes), so it is difficult to make broad generalizations about freshwater systems [3, 21, 22]. The Tingjiang River, which is the largest river in the west of Fujian province, southeastern China, provides the major source of drinking water for a large number of people [23]. This river includes many small tributaries and shows highly dynamic environmental conditions similar to other lotic ecosystems in subtropical monsoon region [3, 24]. Further, the hydropower dams built along the main channel and its tributaries might exacerbate riverine ecosystem vulnerability and reduce the value of ecosystem services [3]. The microeukaryotic diversity and biogeography from the Tingjiang River have not been investigated in the previous studies. Additionally, in aquatic ecosystems, seasonality is a key driver of environmental fluctuation, distinct microbial community diversity, and composition [20]. However, there exists limited knowledge on the factors shaping microeukaryotic biogeography across seasons in subtropical rivers. Therefore, understanding the spatiotemporal patterns, processes, and mechanisms of Tingjiang River’s microeukaryotic community across a hydrologic gradient in different seasons is of great importance because of their importance to river health, water quality, and ecosystem services.

In natural ecosystems, microeukaryotic communities include abundant taxa as well as rare or low-abundance ones [25, 26]. The abundant or dominant taxa normally contribute major functions in ecosystems because of their high abundance [27]. Recent advances in microeukaryotes profiling using the high-throughput sequencing allowed us to directly observe and study the “rare biosphere” [27–29]. Although the dominant taxa drive many ecosystem processes, these rare taxa can also exhibit unique metabolic activities, carrying out particular metabolic functions, and present distinct biogeography [10, 29, 30]. Further, rare and abundant taxa have different life histories that could influence their dispersal capacities [28]. Another interesting point is that taxa which are usually rare but occasionally become more prominent in their communities, namely the conditionally rare taxa (CRT). The rare-to-abundant occurrence pattern of CRT has been reported in multiple ecosystems, where CRT ecology may help to identify the biological, chemical, and physical drivers of microbial dynamics [29]. Considering the ecological importance and difference of always rare taxa (ART), conditionally rare taxa (CRT), and dominant taxa, understanding whether these groups exhibit similar or different biogeography in rivers will provide a more comprehensive understanding for microeukaryotic community assembly [25, 28].

**Fig. 1:**
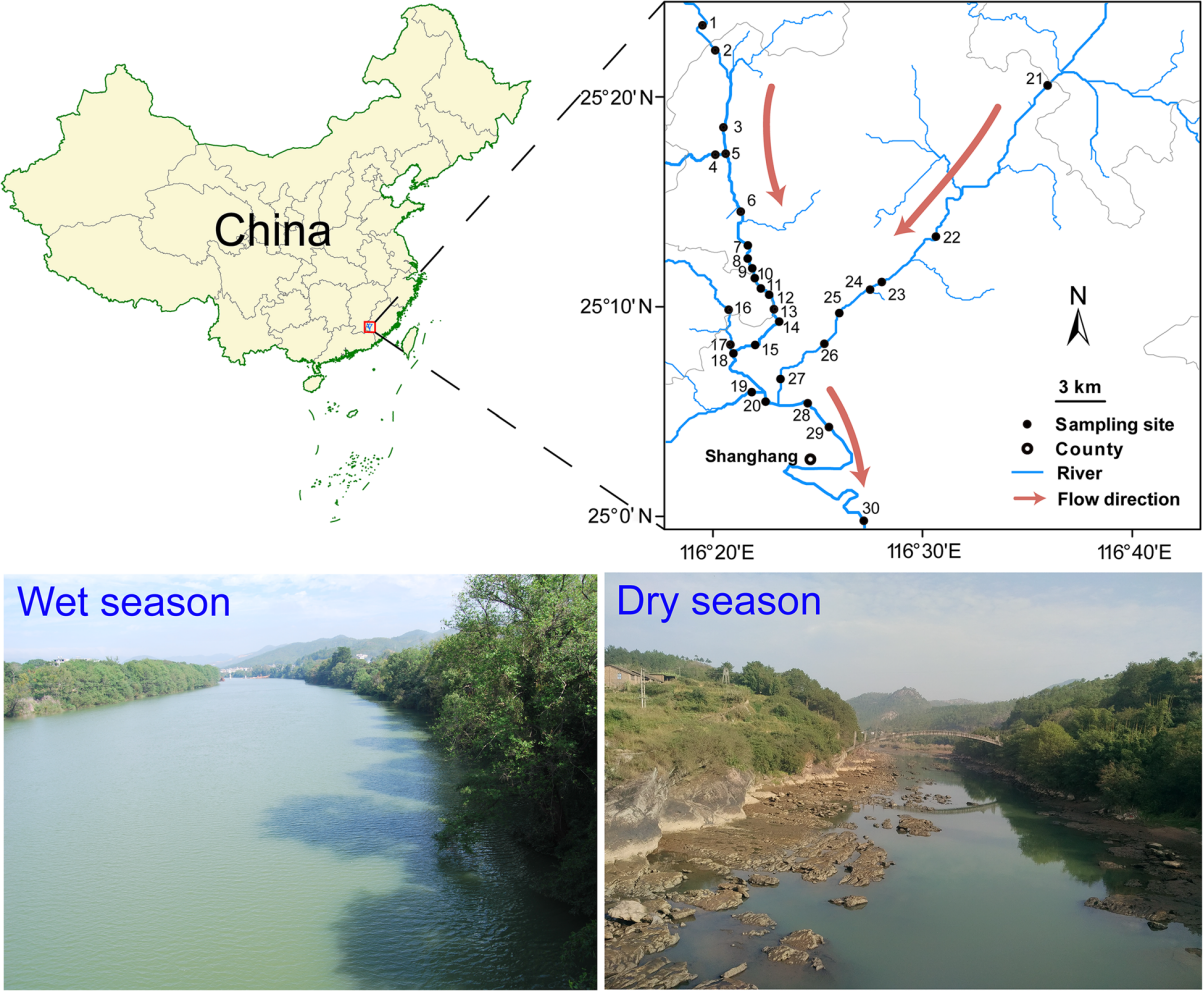
Sketch map of Tingjiang River showing the sampling sites in wet and dry seasons. A total of 60 surface water samples were collected in July and November 2015. The map of Tingjiang River sampling sites was performed using ArcGIS 10.1 (ESRI, Redlands, CA, USA)

In this study, the planktonic microeukaryotes, together with environmental and spatial factors, were analyzed in the Tingjiang River during both wet and dry seasons (Fig. 1). We first hypothesized that microeukaryotic plankton communities exhibit a significant distance-decay pattern during both wet and dry seasons, and this pattern is stronger in the dry than wet seasons due to high level of the spatially structured environmental gradients in dry season, thereby decreasing the relative importance of stochastic processes. Further, we predicted that abundant and rare taxa are assembled via similar mechanisms as they are likely to have similar ecological responses to environmental and spatial changes [5]. In addressing this hypothesis, we separately investigated community assembly mechanisms shaping the entire microeukaryotic communities, as well as the dominant, always rare, and conditionally rare taxa subcommunities. To quantify whether neutral or stochastic processes drive the biogeography, we employed the neutral community model of Sloan et al. [7]. For quantifying the effect of niche or deterministic processes, we examined the relationship between microeukaryotic communities and various environmental and spatial factors using the Mantel test and variation partitioning analysis [3, 20]. Further, given the difference of occurrence frequency and relative abundance among above, within, and below the neutral model predictions, we separated the microeukaryotic taxa into neutral and non-neutral (above and below) fractions, and we expected that these two fractions could show different community compositions with contrasting responses to environmental change. We also hypothesized that the migration rate is a key factor in differentiating neutral and non-neutral fractions. This study aimed to answer the following key questions: (1) Do microeukaryotic communities in wet and dry seasons exhibit similar or different spatiotemporal patterns? (2) Are the total, dominant, always rare, and conditionally rare microeukaryotic taxa assembled via different community assembly mechanisms? (3) How well do neutral processes explain the microeukaryotic assembly across different seasons in the subtropical river? (4) To what extent do species’ migration rate and environmental factors affect neutral and non-neutral community compositions?

## Results

### Comparison of environmental factors between wet and dry seasons

The environmental factors are summarized in Additional file 2: Table S1. Half of the 24 environmental factors showed a significant difference between wet and dry seasons (Additional file 2: Table S2). In general, the mean values of temperature, turbidity, suspended solids, electrical conductivity (EC), TOC, NO_3_-N, and arsenic (As) were significantly higher in the wet season than those of the dry season. On the contrary, pH, dissolved oxygen (DO), total phosphorus (TP), PO_4_-P, and cadmium (Cd) showed higher mean values in the dry season.

### Comparison of microeukaryotic community alpha diversity between wet and dry seasons

Rarefaction curves for individual and combined set of 60 samples showed that most samples from either wet or dry seasons tended to approach saturation, and wet microeukaryotic communities showed higher species richness with the same sequencing effort (Additional file 1: Figure S1). Good’s coverage ranged from 97.68 to 99.31% in the individual samples, and the index of all 60 samples combined was 99.98% (Additional file 2: Table S3). The rarefaction curves, extrapolated species richness indices (ACE and Chao 1), and Good’s coverage indices indicated that the majority of the microeukaryotic taxa had been recovered from the studied metacommunity (Additional file 1: Figure S1 and Additional file 2: Table S3). In general, the microeukaryotic metacommunity in the wet season showed higher alpha diversity compared with those in the dry season.

A total of 17,416 microeukaryotic OTUs were identified from 6,623,640 high-quality sequences at 97% similarity level for the 60 samples, and all microeukaryotic taxa were divided into five categories (conditionally abundant taxa, moderate taxa, always rare taxa, conditionally rare taxa, and conditionally rare and abundant taxa) since no OTU was detected as always abundant taxa (Additional file 2: Table S4).

### Comparison of microeukaryotic community composition between wet and dry seasons

Overall, most diverse OTUs in both wet and dry seasons were assigned to the groups of Stramenopiles, Alveolata, and Fungi, and the OTUs of Stramenopiles were significantly more diverse in the wet than in the dry seasons. In addition, most abundant OTUs were assigned to Alveolata, Animalia, other eukaryota (incertae sedis eukaryota), and Stramenopiles. The proportions of sequences of Alveolata, Animalia, Discoba, Holozoa, and Stramenopiles were significantly higher in wet than in dry seasons, whereas Chloroplastida, other eukaryota, and Fungi showed higher relative abundance in dry than in wet seasons (Additional file 1: Figure S2A).

**Fig. 2:**
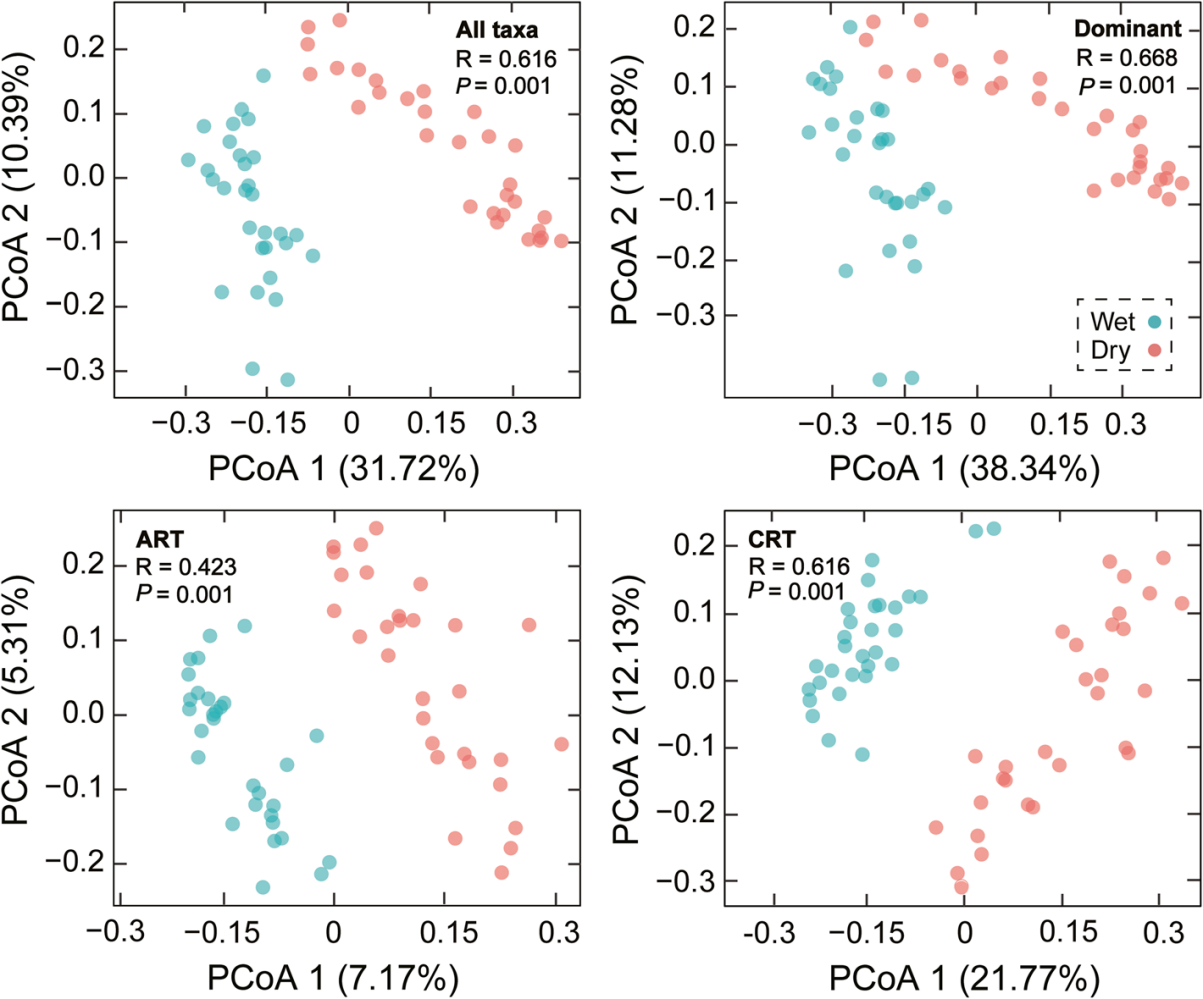
Microeukaryotic community beta-diversity visualized using PCoA ordination based on the Bray-Curtis similarity. Dominant represents taxa including AAT, CAT, and CRAT. AAT, always abundant taxa; CAT, conditionally abundant taxa; CRAT, conditionally rare and abundant taxa; ART, always rare taxa; CRT, conditionally rare taxa

Our PCoA ordinations and ANOSIM tests showed that the microeukaryotic community compositions (including total, dominant, always rare, and conditionally rare taxa) from wet and dry seasons exhibited a clear separation (*R* > 0.423, *P* = 0.001; Fig. 2). Venn diagrams indicated that most OTUs obtained were shared between the two seasons (71.72% for total, 100% for dominant, 64.99% for always rare, and 98.88% for conditionally rare taxa; Additional file 1: Figure S2B). Taken together, there were significant differences in microeukaryotic community composition between wet and dry seasons, although a large proportion of taxa were shared with both seasons in the Tingjiang River.

### Environmental and spatial factors related with microeukaryotic community composition

The Mantel tests indicated that turbidity, electrical conductivity (EC), total carbon (TC), nitrogen (TN and NH_4_-N), phosphorus (TP and PO_4_-P), and arsenic (As) were significantly associated with the microeukaryotic communities (including total, dominant, always rare, and conditionally rare taxa; *P* < 0.05, Additional file 2: Table S5) in the wet season, while water temperature, turbidity, suspended solids, carbon (TOC), nitrogen (TN, NH_4_-N, NO_2_-N, and NO_3_-N), total phosphorus (TP), zinc (Zn), and arsenic (As) were significantly correlated to the microeukaryotic communities in the dry season (*P* < 0.05, Additional file 2: Table S5).

**Fig. 3:**
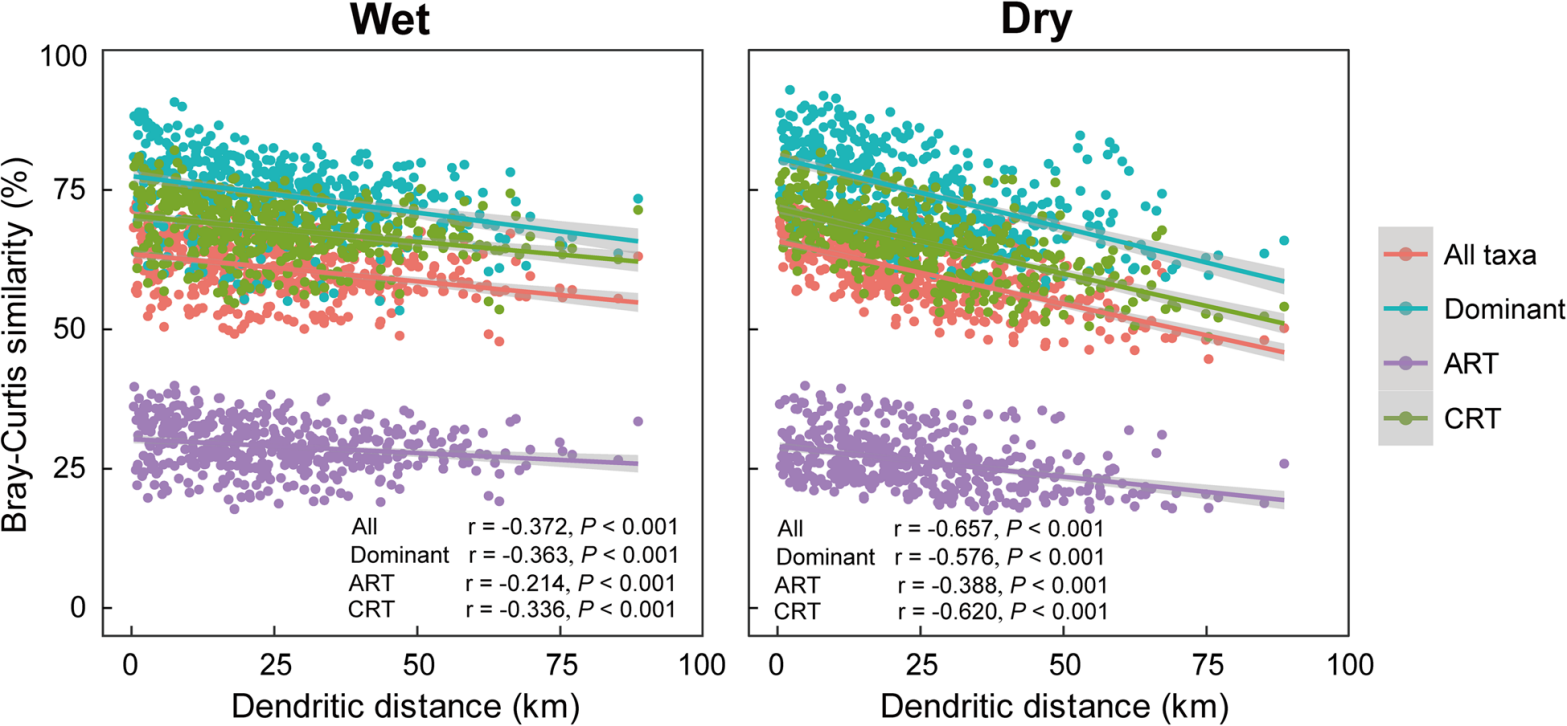
Distance-decay patterns based on the Bray-Curtis similarity of microeukaryotic community composition and cumulative dendritic distance in wet and dry seasons, respectively (*n* = 435). Dominant represents taxa including AAT, CAT, and CRAT. AAT, always abundant taxa; CAT, conditionally abundant taxa; CRAT, conditionally rare and abundant taxa; ART, always rare taxa; CRT, conditionally rare taxa. The shaded area around the lines covers 95% confidence interval of the correlations

The two different spatial predictors (dendritic distance and Euclidian distance between sampling sites) of microeukaryotic community compositions showed similar results (Fig. 3, Additional file 1: Figure S3, and Additional file 1: Figure S4). There were significant and negative relationships between the geographical distance and Bray-Curtis similarity of microeukaryotic communities (including total, dominant, always rare, conditionally rare taxa) in both wet and dry seasons (*P* < 0.01; Fig. 3, Additional file 1: Figure S3). Further, the biogeography of both the always rare taxa and conditionally rare taxa was similar to that of the dominant and total microeukaryotic communities. Additionally, Spearman’s rank correlations between the Bray-Curtis similarity of river microeukaryotes (from genus to kingdom levels) and two types of spatial distances (dendritic and Euclidian distances) also showed a significant distance-decay relationship (Additional file 1: Figure S4). All of the microeukaryotic subcommunities exhibited a robust distance-decay pattern, whereby community dissimilarity increased with geographical distance, indicating that these distinct microeukaryotic taxa under different hydrologic regimes (i.e., wet and dry seasons) exhibited general similar spatial pattern. Further, among the correlations between spatial distances (either dendritic or Euclidian) and microeukaryotic communities, all of the microeukaryotic communities exhibited a stronger distance-decay pattern in the dry season than in the wet season (Fig. 3, Additional file 1: Figure S3, and Additional file 1: Figure S4).

### Relative contribution of environmental and spatial factors on microeukaryotic distribution

The variance partitioning analyses (VPA) results showed that only a small proportion of microeukaryotic community variation was explained by environmental and spatial factors (Additional file 2: Table S6, Additional file 2: Table S7, and Additional file 2: Table S8). Both variance partitioning analyses (VPA) using environmental vs. principal coordinates of neighboring matrices (PCNM), and environmental vs. asymmetric eigenvector map (AEM) variables revealed similar results in both the wet and dry seasons. The principal coordinates of neighboring matrices (PCNM) analysis, represents the geographical distance matrix as Euclidean distance between each pair of sampling sites, while the asymmetric eigenvector map (AEM) analysis represents the network distance matrix between two sites along the river.

Our VPA performed on environmental model and directional spatial model (AEM) showed that over the four communities (total, dominant, always rare, and conditionally rare taxa) in the wet season, the pure environmental variables (e.g., temperature, pH, As), the pure spatial processes (the spatial structuring of the microeukaryotic community characterized by AEM variables), and their shared effect explained mean 5.6%, 11.7%, and 8.2% of the community variations, respectively. For dry season, the pure environmental, the pure spatial variables, and the shared effect explained mean 7.9%, 18.2%, and 2.6% of the community variations, respectively. Consequently, a large amount of the variance (on average 74.5% for wet season and 71.3% for dry season, respectively) remained unexplained by the environmental and spatial variables (Additional file 2: Table S6). The VPA based on environmental and PCNM variables (Additional file 2: Table S7 and Additional file 2: Table S8) revealed similar results with VPA based on environmental and AEM variables. The high proportion of unexplained variation in microeukaryotic taxa in wet and dry seasons indicated the potential importance of neutral or stochastic processes for community assembly, especially in wet season.

### Fit to the neutral model of community assembly

**Fig. 4:**
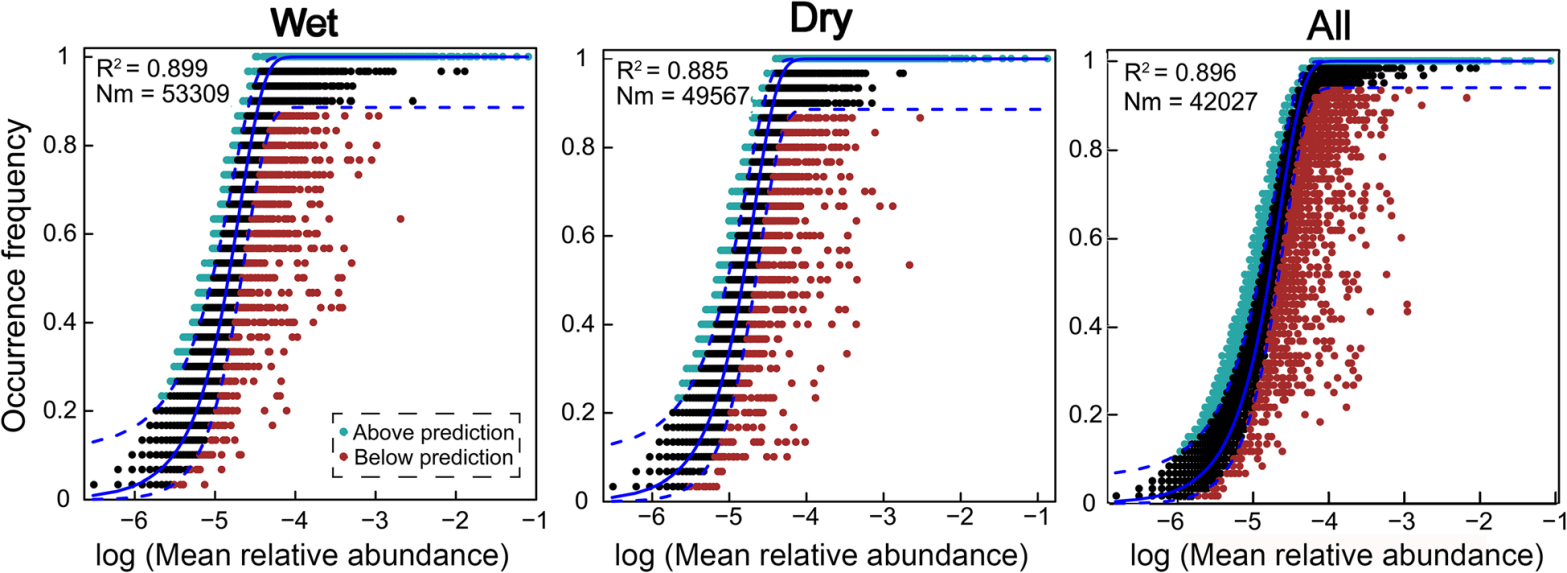
Fit of the neutral community model (NCM) of community assembly. The predicted occurrence frequencies for wet, dry, and all representing microeukaryotic communities from wet, dry, and both seasons, respectively. The solid blue lines indicate the best fit to the NCM as in Sloan et al. [7], and the dashed blue lines represent 95% confidence intervals around the model prediction. OTUs that occur more or less frequently than predicted by the NCM are shown in different colors. *Nm* indicates the metacommunity size times immigration, *R*^2^ indicates the fit to this model

The neutral community model (NCM) successfully estimated a large fraction of the relationship between the occurrence frequency of OTUs and their relative abundance variations (Fig. 4), with 89.9%, 88.5%, and 89.6% of explained community variance for wet, dry, and both seasons, respectively. Further, the NCM of microeukaryotic community at supergroup levels showed large explained community variance for wet (ranged from 78.8 to 99.3%, with mean value 91.3%) and dry seasons (varied from 67.5 to 97.0%, with mean value 88.8%), respectively (Additional file 1: Figure S5).

More importantly, the explained variation of NCM tended to remain relatively large and constant along taxonomic ranks from genus to kingdom taxonomic resolutions (all of the *R*^2^ over than 88.8% with mean value 92.7%), indicating that taxa within the same phylogenetical lineages generally show similar responses to stochastic processes (Additional file 1: Figure S6). These results indicated that stochastic processes were very important in shaping the microeukaryotic community assembly in both seasons. The *Nm*-value was higher for microeukaryotic taxa in the wet season (*Nm* = 53,309) than the dry season (*Nm* = 49,567). Since the number of sequences in both samples was 110,394, the *m* value was estimated to be 0.483 in wet season and 0.449 in dry season, respectively. These results indicated that species dispersal of planktonic microeukaryotes was higher in the wet than dry seasons.

### Neutral and non-neutral partitions are ecologically distinct

**Fig. 5:**
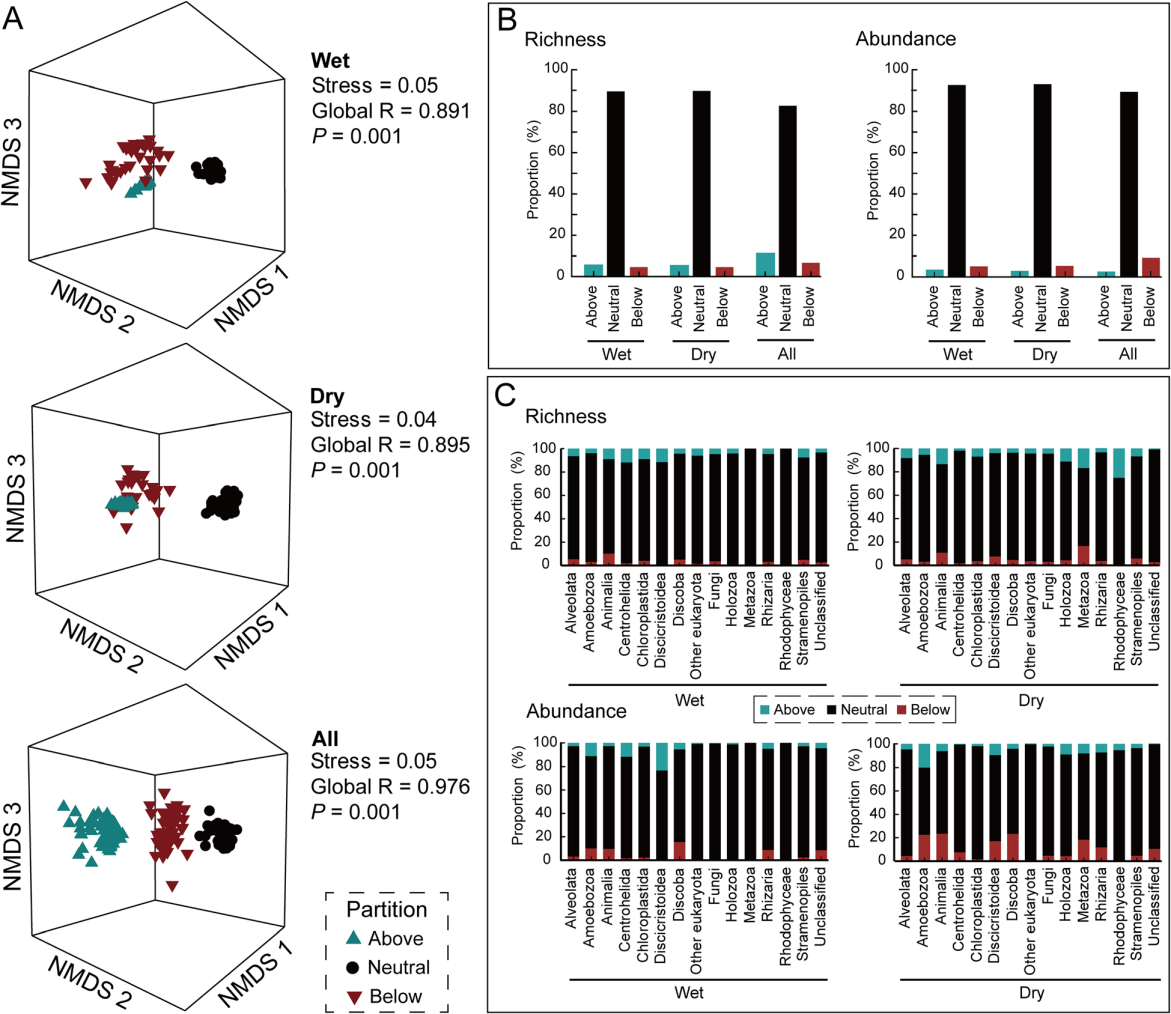
Neutral and non-neutral partitions of the metacommunity showed distinct community composition, diversity, and abundance. For each season group, communities were pooled and OTUs were then divided into separate partitions based on whether they were consistent within (in black) or deviated above (in light blue) or below (in dark red) the neutral prediction (color coding is consistent for all panels). **a** Non-metric multidimensional scaling ordination based on the Bray-Curtis similarity. **b** The distinct fitting proportions of microeukaryotic communities’ OTU and sequence numbers by the Sloan neutral model. **c** The distinct fitting proportions of richness (OTU number) and abundance (sequence number) of wet and dry seasons’ microeukaryotic supergroups. The other eukaryota represent incertae sedis eukaryota. The wet, dry, and all representing microeukaryotic communities from wet, dry, and both seasons, respectively

For any season, there were a number of microeukaryotic taxa that occurred more or less (above or below neutral prediction) frequently than predicted by NCM given their overall abundance in the metacommunity (Fig. 5). We would consider the taxa that differ significantly from the neutral prediction were due to their different migration or dispersal ability. Points above the prediction represent taxa that are found more frequently than expected, suggesting that they have higher migration ability and can disperse to more locations. Points below the prediction represent taxa found less frequently than expected, suggesting their lower dispersal ability in the river (Additional file 2: Table S9). Another possibility is that this could be due to taxa responding more strongly to local environmental conditions (Additional file 2: Table S10). Partition fractions above the neutral prediction were mainly composed of Centrohelida, other eukaryota, and Holozoa (the estimated migration rate over than 0.6), while the main supergroups of below partition were Animalia, Discoba, and Rhodophyceae (the estimated migration rate less than 0.3, Additional file 2: Table S11).

Taxa from neutral and non-neutral fractions (above and below neutral prediction) showed contrasting community composition, diversity, and abundance (Fig. 5). For wet, dry, and both seasons, we separated the metacommunity into three fractions comprising those OTUs found above, below, and neutral partitions, and analyzed the non-metric multidimensional scaling ordination (NMDS) based on the Bray-Curtis similarity among partitions. The results clearly demonstrated that above, below, and neutral partitions of taxa showed significantly different compositions among wet (global *R* = 0.891), dry (global *R* = 0.895), and both (global *R* = 0.976) seasons at *P* < 0.01 (Fig. 5a). Further, non-neutral fractions of the metacommunity were also much more heterogenous than the neutral fractions. In addition, the proportions of microeukaryotic OTUs and sequence numbers at supergroup level showed that the neutral fraction accounted for much higher richness and abundance proportions than above and below fractions in both wet and dry seasons (Fig. 5b, c).

Neutral and non-neutral partitions’ microeukaryotic community compositions showed different responses to environmental and spatial factor change. The Mantel test between environmental factors and three microeukaryotic community partitions predicted by the NCM showed that neutral and non-neutral partitions of the metacommunity were significantly associated with both common and different environmental variables in wet and dry seasons. For example, EC and As in the wet season and nitrogen (NH_4_-N, NO_3_-N, and NO_2_-N) and As in the dry season were significantly correlated to both the neutral and non-neutral partitions’ microeukaryotic communities. However, other environmental factors such as water temperature, suspended solids, current velocity, TN, NO_2_-N, TP and Cd in the wet season, and water temperature, PO_4_-P and Zn in the dry season were only significantly associated with the non-neutral partition. Further, turbidity was only significantly correlated to neutral partition in the dry seasons (*P* < 0.05, Additional file 2: Table S10). The neutral and non-neutral partitions’ microeukaryotic taxa also showed distinct degrees of distance-decay patterns, although all of the relationships between microeukaryotic community and geographical distance were significant. And the relationship was stronger in the dry than wet seasons (the absolute *r* value of Mantel tests between microeukaryotic community and geographical distance was higher in the dry than wet seasons; Additional file 2: Table S10).

## Discussion

### Spatiotemporal patterns of microeukaryotic communities in river ecosystem

The biogeography of microeukaryotic plankton across different hydrographic regimes has received little attention so far in river ecosystems [24]. Our study contributes to understanding the spatiotemporal patterns of microeukaryotic plankton and sheds light into the underlying processes and mechanisms. As expected, the PCoA analysis illustrated that microeukaryotic plankton communities from the same season clustered together (Fig. 2), indicating that microeukaryotes from wet and dry seasons exhibited distinct community compositions. Several reasons may explain this phenomenon. First, this result might be because of the variation in environmental and hydrographic conditions between the two seasons [3]. The difference in certain environmental factors (e.g., temperature, turbidity, and pH) between the two seasons was significant in this study (Additional file 2: Table S2). Second, the seasonal succession of freshwater plankton including microbes is an annually repeated process due to various external factors and internal interactions, such as physicochemical factors and species interactions [31]. Third, the distinct community distribution from different studied stations suggests that stochastic processes (e.g., dispersal and drift) influenced microeukaryotic community assembly [2, 15]. More importantly, downstream flow of the river can cause high level of microbial dispersal, especially in the wet season, which leads to much lower beta diversity (i.e., homogenization of the plankton communities, as shown in the PCoA) in wet than dry seasons (Fig. 2). Further, the lotic systems have an unusually large boundary with other ecosystems, such as surrounding soil and leaves in the watershed. The rain events might wash the microbes from surrounding systems, which also lead to distinct community diversity, compositions, and increase microbial community variations in wet season. Therefore, the interaction among different environmental, biotic, and spatial conditions could shape the distinct community composition along the Tingjiang River [15, 31, 32].

### Similar biogeography of dominant, always rare, and conditionally rare microeukaryotes

Previous study has showed that some rare taxa were metabolically active in the environment, and they may be keystone species in regulating the functioning of aquatic habitat [28]. So the “rare biosphere” is of great importance to metabolic and ecological functions of aquatic ecosystems [25, 27, 28]. Conditionally rare taxa (CRT, the taxa expected to be usually rare under certain environments and occasionally achieve prevalence when conditions become adequate) have been reported that they can explain large temporal shifts in the microbial communities structure [29]. However, our knowledge of many ecosystems is mostly based on the dominant and entire communities, with the role of always rare taxa (ART) and CRT remaining unaddressed. For example, what mechanism is underlying the assembly of always rare and conditionally rare microeukaryotic taxa across hydrographic regimes in subtropical river? How does the biogeography of always rare and conditionally rare microeukaryotic community differ from that of dominant and entire taxa?

Our data indicated that the ART and CRT exhibited similar biogeography with dominant and entire communities in both wet and dry seasons (Fig. 3). The biogeographical patterns for the rare biosphere clearly showed that the dictum “everything is everywhere” does not apply here [30]. It therefore suggested that river microeukaryotes were not cosmopolitan distribution as previously thought, and so barriers for dispersal existed on the rare biosphere. This could be because rivers close to each other tend to have similar environmental condition. It also suggested that these groups have comparable environmental and spatial sensitivity, and they responded to environmental and spatial change in a similar manner [32]. The observation of similar biogeographic patterns of rare and dominant taxa was consistent with previous study from the bacterioplankton communities in coastal Antarctic lakes [32], but contrasted to the study focused on bacterial taxa in an activated sludge bioreactor [27]. These differences might have resulted from different environmental settings and spatial gradients in these studies. The first two studies were carried out in natural environments (river and lake) across space, while later one was operated in artificial environment across time. Rare taxa in natural environments might more likely exhibit a similar biogeography to dominant taxa.

As expected, the distance-decay pattern was stronger during the dry than wet seasons in Tingjiang River (Fig. 3; Additional file 1: Figure S3 and Additional file 1: Figure S4; Additional file 2: Table S10). This was not surprising because microeukaryotic planktons face less dispersal limitation in wet season. In contrast to macroorganisms, microbes are dispersed more easily due to their smaller size and they are unable to counteract unidirectional movement of the water flow. Rainfall always concentrates in the summer (wet season) in the subtropical rivers of China [24] and can cause high water level and high river flow. The increase of river flow can enhance habitat homogeneity and river connectivity [3, 20], and the microbes can more easily and passively disperse with water flow to distant locations in wet season. On the contrary, rain events are very rare in the dry season, leading to a lower river flow and higher dispersal limitation of microbes (Fig. 1). Further, the dams might result in decreases of flow rate, which are particular noteworthy in dry season [20]. The dams can lead to the shortage of water in the lower reaches of the river and decrease river connectivity in the dry season, therefore promoting the heterogeneity in microhabitats of microeukaryotic communities.

### Minor influence of environmental and spatial factors on the microeukaryotic community

Although several environmental variables were identified as having a significant effect on the microeukaryotic community compositions, our VPA results indicated that environmental and spatial factors play a minor role in shaping the communities, as revealed by a small proportion of community variation explained by these two factors. Especially for the ART, > 90% of community variation remained unexplained by space and the environment in wet and/or dry seasons (Additional file 2: Tables S6–S8). Several previous studies also found large proportion of unexplained rare bacterioplankton and microeukaryotic community variations in different habitats and sampling areas using VPA [5, 13, 33]. Although VPA is widely used in ecological research to determine the relative importance of environmental selection and spatial effects on microbial community structure, some studies have shown that it is not a valid method to infer the influences of ecological processes [34, 35]. Several reasons could account for this result. First, the large unexplained community variation in this study could be explained by the absence of important factors taken into account in the VPA [36–38]. Second, other studies showed that the co-occurrence correlations among microbes can influence the community distribution, which cannot be quantificated by VPA [9, 11]. Third, the VPA tends to undervalue the contribution explained by environmental variables [34], which might also be a possible reason for the low contribution of deterministic or selective processes in shaping the microeukaryotic community variation. Therefore, great caution is needed when using VPA to partition community variation, and it should be used as an exploratory tool together with other approaches to develop hypotheses and determine the relative importance of environmental and spatial variables. In this study, we used neutral community model to estimate the relative importance of stochastic processes. This method does not relate community composition to environmental and spatial variables and therefore can overcome the shortcoming of VPA.

### Microeukaryoic community assembly mainly shaped by stochastic processes

Our results clearly support the prominent role of stochastic processes in shaping the microeukaryotic community assembly (Fig. 4, Additional file 1: Figure S5, and Additional file 1: Figure S6). The neutral community model (NCM) is a neutral-based process model, which is a valid approach for inferring stochastic processes acting on community assembly, and has been successfully applied to a wide range of ecological phenomena [4, 39]. This model allows researchers to quantify the importance of processes which are difficult to observe directly but can have large influence on microbial communities (i.e., dispersal and ecological drift) [40]. The NCM estimated a major part of the community variation (including different supergroup levels’ and taxonomic resolutions’ taxa) suggesting that stochastic balance between the loss and gain of microeukaryotes (such as stochastic births, deaths, and immigration) were critical in shaping their community assembly (Fig. 4; Additional file 1: Figure S5 and Additional file 1: Figure S6) [1, 6, 7]. The significantly strong distance-decay pattern of plankton communities during wet and dry seasons further confirmed the importance of stochastic processes (Additional file 1: Figure S3 and Additional file 1: Figure S4), since according to Hubbell’s neutral theory [6], community similarity was predicted to decrease along spatial (distance) gradients due to dispersal limitation [41]. Several key observations also revealed similar results to our finding. For example, Roguet et al. [15] investigated the bacterial community in 49 lakes of Paris area, France, and showed that bacterial community structure was mainly driven by stochastic processes (*R*^2^ = 0.76 explained by NCM). In addition, Östman et al. [14] revealed that the NCM explained about 85% of detection frequency for aquatic bacteria from the lakes, streams, or rock pools. The stochastic processes are powerful enough to generate a large amount of community diversity both within and among different seasons along the river.

The value of NCM parameter *R*^2^ was slightly higher in the wet than dry seasons, and according to the calculated *Nm* values, plankton dispersal between the sampling sites in wet season is likely higher than dry counterparts (assuming similar metacommunity sizes, Fig. 4), indicating that the influence of stochastic processes was stronger for the microeukaryotes in the wet season [7]. Further, regarding the microeukaryotic community immigration rate, the *m* values in wet season were higher compared with dry season (Fig. 4; Additional file 2: Table S11), indicating the dispersal ability of most microeukaryotic taxa in wet season was higher than dry season counterparts. These results might be attributed to the higher habitat homogeneity and river connectivity in wet season compared with dry season, as showed by the weaker distance-decay relationship in wet season (Fig. 3). The rainfall events and high river connectivity can increase the possibility of the movement and successful establishment of microorganisms across space, leading to higher microeukaryotes’ immigration rate in wet season. High dispersal rate can partly overwhelm both environmental selections and ecological drift. Together with the relatively minor spatial influence on communities, the wide distribution range of plankton suggests that dispersal limitation has only a weak effect on spatial turnover in microeukaryotic communities during wet season.

Although the NCM had a good fit to microeukaryotic data, it is difficult to infer the absence of the influence of environmental and spatial variables on microeukaryotic community composition [42], especially as the large proportion of unexplained variance revealed by VPA, which could be ascribed to drift, unmeasured environmental variables and species interactions [4, 16]. Some of key environmental variables can change in a stochastic manner, because river ecosystems are extremely dynamic, and snapshot sampling normally includes a large fraction of noise, which may mask the main ecological patterns. Moreover, in our study, we did not identify any other important mechanisms except the stochastic processes that could explain the microeukaryotic community distribution, such as mass effect (which assumes that massive immigration can rescue microbes from competitive exclusion, thus relaxing the connection between community composition and local environmental conditions), which cannot be clearly identified by either VPA or NCM [36], but appears to have a strong effect on microeukaryotic community composition in oligotrophic lakes [43]. Further, the co-occurrence correlations among microbes should also be responsible for the community structure [9, 11]. Microbial communities of different taxonomic and functional groups may be structured by contrasting underlying factors [8]. To fully understand the mechanisms of microeukaryotic community assembly in subtropical rivers, we need further experimental consideration and more effective statistical analyses through space and time.

### Difference between neutral and non-neutral partitions

The NCM did not explain 100% of the microbial community variation, indicating that other community assembly mechanisms might be existing at the same time, which caused the non-neutral distribution. These other mechanisms include environmental selection and species interactions [4, 44], which remain difficult to quantify their relative contribution on microeukaryotes. The NCM separated taxa into neutral and non-neutral partitions, and these two fractions were formed of different richness and abundance, leading to different community compositions (Fig. 5). It is possible that non-neutral behavior in these communities is due to the different dispersal rates among taxa (Additional file 2: Table S9). The above partitions’ taxa (e.g., some species of Holozoa) had higher immigration abilities than neutral and below partitions, leading to higher occurrence frequency (Fig. 4; Additional file 2: Table S11). Therefore, partitioning the communities according to the different immigration rates would likely improve neutral predictions. The migration rate reflects the dispersal ability of taxa, and it can represent an important indicator of NCM. A previous study using NCM predicted the community assembly of microbiota associated with the intestine of the zebrafish over host developmental time, and they found that estimated migration rates decreased over host development, which showed consistent variation tendency with the fit of NCM [39]. These results indicated that higher dispersal rate caused better fit of this model in wet season. Further, another study showed that stochastic forces were important in driving the skin fungal community assembly, and also found that taxa in the above-neutral, below-neutral, and neutral partitions formed three distinct clusters across four seasons (winter, spring, summer, and autumn in a calendar year) [40]. The differences between clusters were driven by a small number of taxa which possessed distinct physiological functions [40]. Additionally, except for migration rate (the dispersal of stochastic processes), other factors such as environmental factors (deterministic processes) also can separate the taxa into neutral and non-neutral partitions in this study. Our Mantel test showed that neutral and non-neutral partitions of the metacommunity were significantly associated with some distinct environment factors during wet and dry seasons, respectively (Additional file 2: Table S10). The environmental factors (i.e., water temperature, nutrient concentrations, and heavy metal) have been previously demonstrated to have significant influence on the microbial community composition [5, 21, 24, 32], then affecting the relative abundance and occurrence frequency of planktonic microorganisms, thereby resulting in the neutral and non-neutral distribution. Other factors which also can separate the taxa into neutral and non-neutral partitions need further study in the future.

## Conclusions and implications

This study provides a better understanding of spatiotemporal patterns, processes, and mechanisms underlying the microeukaryotic community and reveals the importance of the stochastic processes on the microeukaryotic metacommunity assembly in a subtropical river across different hydrographic regimes. It demonstrated that different seasons had distinct microeukaryotic community compositions (including total, dominant, always rare, and conditionally rare taxa). Some environmental and spatial factors could significantly influence microeukaryotic distribution, but they only played a minor role in shaping the microeukaryotic communities. The always rare and conditionally rare taxa exhibited similar biogeography with dominant and entire microeukaryotic communities in wet and dry seasons, suggesting the biogeography of the rare biosphere argued against a cosmopolitan distribution, and rather inferring ecological controls of limited dispersal potentials. Further, the distance-decay relationship was stronger during the dry than wet season as expected. For both wet and dry of microeukaryotic groups, stochastic processes strongly shaped microeukaryotic community compositions, whereas deterministic factors played a minor role in influencing community distribution during either wet or dry seasons. Our NCM prediction indicated that neutral and non-neutral partitions (above and below neutral prediction) showed contrasting community composition, diversity, and abundance, and perhaps, migration rate was an important indicator to separate these two partitions. Although the NCM successfully predicted the microbial community distribution, it is difficult to infer the large proportion of unexplained variance revealed by VPA. To fully understand the microeukaryotic community assembly mechanisms, it is suggested that future microbial community ecology researches should consider the sampling scale effects (spatial extent and time scale), more important explanatory deterministic factors (e.g., unmeasured environmental factors and species interactions), and other possible stochastic factors.

## Materials

### Study area, sampling, and environmental factors

This study was carried out in 30 stations along the Tingjiang River (116° 19′–116° 37′ E, 24° 59′–25° 24′ N) in Shanghang county, Fujian province, southeast China (Fig. 1). Given the variable nature of river planktonic communities, a comprehensive investigation of the role of stochastic processes in shaping the communities requires a high degree of replication and control. A total of 30 surface water samples (0.5 m depth) were collected in July and November 2015, representing the wet and dry seasons, respectively. The samples were collected within a 5-day period in both seasons. After collection, these samples were transported to the laboratory and processed immediately. For microeukaryotic community analyses, water samples were pre-filtered through a 200-μm pore-size sieve to remove debris, mesoplankton, and macroplankton, and then, each water sample (~ 500 ml) with microeukaryotes (smaller than 200 μm) was subsequently filtered through a 0.22-μm pore-size polycarbonate membrane (47 mm diameter, Millipore, Billerica, MA, USA). The membranes were stored at − 80 °C until DNA extraction.

The latitude and longitude for the studied sites were determined by a portable global positioning system (GPS Jisibao G330, Bejing, China). All physicochemical analyses were measured according to methods used in our previous study [24]. Briefly, a Hydrolab DS5 multiparameter water quality meter (Hach Company, Loveland, CO, USA) was used to measure water temperature, dissolved oxygen (DO), electrical conductivity (EC), salinity, pH, oxidation reduction potential (ORP), chlorophyll-*a* (Chl-*a*), and turbidity in situ. The current velocity was measured with a SonTek flowtracker (YSI, San Diego, CA, USA). Suspended solids were gravimetrically measured by filtering 100 ml water samples through pre-weighed 0.45-μm pore-size filters; these filters were reweighed again after evaporation at 105 °C. Total carbon (TC), total organic carbon (TOC), and total nitrogen (TN) were analyzed with a TOC/TN-VCPH analyzer (Shimadzu, Tokyo, Japan), and total phosphorus (TP) was determined using spectrophotometry according to the standard methods. The concentrations of ammonium nitrogen (NH_4_-N), nitrite and nitrate nitrogen (NOx-N), and phosphate phosphorus (PO_4_-P) were determined following our previous study [24]. Further, arsenic (As) and heavy metals (Cr, Cu, Zn, Cd, Hg, and Pb) were determined using an inductively coupled plasma mass spectrometry (Agilent Technologies Inc., Bellevue, WA, USA), as previously reported [45]. In total, 24 environmental variables were measured in this study.

### DNA extraction, PCR and Illumina sequencing

The DNA of microeukaryotic plankton was extracted directly from the membrane using a FastDNA spin kit (MP, Biomedicals, Santa Ana, CA, USA) following the manufacturer’s instructions. The hypervariable V9 region of eukaryotic 18S rRNA gene was amplified using the forward primer 1380F and reverse primer 1510R [46]. The 30-μl PCR mixture contained 15 μl of Phusion Master Mix (New England Biolabs, Beverly, MA, USA), 0.2 μM of forward and reverse primers, and 10 ng of the sample DNA. PCR reactions included an initial denaturation at 98 °C for 1 min, followed by 30 cycles of 10 s at 98 °C, 30 s at 50 °C, and 60 s at 72 °C. Finally, the amplicons were subjected to final 10 min extension at 72 °C.

Triplicate PCR products for each of 60 samples were conducted and purified using GeneJET Gel Extraction Kit (Thermo Scientific, Hudson, NH, USA). Both negative and positive controls were used through the experiment. Sequencing libraries were generated using the NEB Next Ultra DNA Library Prep Kit for Illumina (New England Biolabs, Beverly, MA, USA) according to manufacturer’s instructions, and index codes were added. The library quality was evaluated using the Agilent Bioanalyzer 2100 system (Agilent Technologies Inc., Bellevue, WA, USA) and Qubit 2.0 Fluorometer (Thermo Fisher Scientific, Waltham, MA, USA). Finally, the bar-coded amplicons from each of samples were mixed in equimolar amounts and then were sequenced using an Illumina Miseq platform (Illumina Inc., San Diego, CA, USA) following the manufacturer’s protocols [47].

### Bioinformatics

Sequenced paired-end reads were merged with FLASH [48]. Raw data were processed and analyzed using QIIME v.1.8.0 to remove low-quality reads [49]. Sequences were quality controlled with the following settings: maximum number of consecutive low-quality base = 3, minimum of continuous high-quality base = 75% of total read length, ambiguous bases > 0 were removed, and last quality score = 3 [26]. Chimeras were identified and removed using UCHIME before the downstream analyses [50]. After that, sequences were clustered into OTUs using UPARSE [51] with the 97% sequence similarity cutoff. Representative sequence from each OTU was aligned against the SILVA (Release 123) reference alignment using the RDP classifier [52]. To avoid mistake in the taxonomic assignments, the taxonomic classifications were checked and followed the reference of eukaryotes [53]. Because of high abundance of unidentified taxa in public databases, some OTUs were treated as unclassified microeukaryotes. Unassigned OTUs (sequence similarity to a reference sequence is < 80%) and singletons (OTUs with only one sequence) were discarded prior to further analysis. Finally, to minimize biases associated with sequencing coverage and allow for comparison of community pattern among 60 samples, the sequence data were normalized to 110,394 sequences per sample.

### Definition of abundant and rare taxa

In this study, we defined relative abundance thresholds as 0.01% for rare taxa and 1% for abundant taxa and classified all OTUs into six categories (AAT, CAT, MT, ART, CRT, CRAT) according to the recent publications [10, 13, 54]. The following exclusive categories were shown in Additional file 1: Figure S7: (1) always abundant taxa (AAT) were defined as the OTUs with abundance ≥ 1% in all samples; (2) always rare taxa (ART) were defined as the OTUs with abundance < 0.01% in all samples; (3) moderate taxa (MT) were defined as OTUs with abundance between 0.01 and 1% in all samples; (4) conditionally rare taxa (CRT) were defined as with abundance below 1% in all samples and < 0.01% in some samples; (5) conditionally abundant taxa (CAT) were defined as taxa with abundance ≥ 0.01% in all samples and ≥ 1% in some samples but never rare (< 0.01%); and (6) conditionally rare and abundant taxa (CRAT) were defined as OTUs with abundance varying from rare (< 0.01%) to abundant (≥ 1%). In this study, we artificially combined abundant taxa (AAT), conditionally abundant taxa (CAT), and conditionally rare and abundant taxa (CRAT) as abundant taxa to perform further analyses, and these three pooled categories (AAT, CAT, and CRAT) were called “dominant taxa” to avoid confusion [13].

## Statistical analyses

### Alpha-diversity analysis

Venn diagrams and alpha diversity indices including OTU richness, ACE (abundance based coverage estimator), Chao 1, Shannon-Wiener, Pielou’s evenness, and Simpson’s index were calculated for each sample in vegan 2.4-1 with R software (version 3.2.3). Rarefaction curve and Good’s coverage were performed in MOTHUR v.1.33.3 [55]. One-way analysis of variance (ANOVA) and Student’s *t* test were used to compare alpha-diversity by SPSS 22.0 (IBM Corp., Armonk, NY, USA).

### Beta-diversity analysis

The Bray-Curtis similarity matrix is considered to be one of the most robust similarity coefficients for ecological studies [56] and was applied to our microeukaryotic community datasets. In this study, the principal coordinate analysis (PCoA) was employed using the Bray-Curtis similarity matrices for detecting differences in wet and dry seasons’ microeukaryotic communities.

The non-metric multidimensional scaling analysis (NMDS) was used to evaluate changes in microeukaryotic composition between neutral and non-neutral (above and below) partitions using PRIMER v.7.0 (PRIMER-E, Plymouth, UK) [57]. Analysis of similarities (ANOSIM) was used to evaluate the significant differences between groups. The statistic global *R* represents the separation degree of between-group and within-group mean rank similarities. *R* = 0 indicates no separation, whereas *R* = 1 indicates complete separation [57].

### Correlation of microbial communities with environmental factors and geographical distance

To explore the potential controlling factors for the microeukaryotic community composition, we used the Mantel tests to reveal the correlations between the community similarity and environmental factors. The environmental factors except pH were square root transformed, and Euclidean distances between sampling sites were calculated. To explore the spatial predictor of microeukaryotic community composition, two contrasting measurements that represented site location were used (Additional file 1: Figure S8). These were (1) the dendritic network length (km), which is a measure of the cumulative length of the branching river network (watercourse) of two sampling sites, and (2) the Euclidian distance (km) based on the longitude and latitude coordinates of each sampling site, which is simply the straight line distance between sampling points. All geographical measures were calculated using ArcGIS (ESRI, Redlands, CA, USA). Spearman’s rank correlations were used to determine the relationships between the Bray-Curtis similarity of microeukaryotic community, the Euclidean distance of environmental factors, and the geographical distance of sampling sites, respectively.

### The relative contribution of environmental and spatial factors in microbial assembly processes

We further used variation partitioning analysis (VPA) with adjusted *R*^2^ coefficients based on redundancy analysis (RDA) and partial Mantel test to quantify the relative effects of environmental and spatial factors in shaping community composition [10, 13]. To explore the better spatial predictor of microeukaryotic community composition, two types of distance matrices were calculated: (a) the geographical distance matrix as Euclidean distance between each pair of sampling sites, which was a set of spatial variables that were generated through the method of principal coordinates of neighboring matrices (PCNM) analysis based on the longitude and latitude coordinates of sampling sites, and (b) the network distance matrix, which was calculated to model the least-cost dispersal route between two sites along the river network. We applied asymmetric eigenvector map (AEM) analysis, which was specifically designed to model directional patterns [58], to generate spatial variables along directional flow. A site-by-edge binary matrix was constructed based on coordinates of the sites and the directional links (edges) among sites using the *build.binary* function in R package *AEM*. Thereafter, the following weighting function were applied: weight = 1 − (*d*/*d*_max_)^2^, where *d* is the network distance between linked sites and *d*_max_ was the maximum distances among value *d* [59] and was assigned to each edge. Finally, the *aem* function in R package *AEM* was used to create eigenvectors [58].

Subsequently, variance inflation factors (VIFs) were calculated to check the collinearities among environmental vs. PCNM (based on partial RDA and partial Mantel test) and environmental vs. AEM (based on partial RDA) variables using the “vegan” package, and variables with VIF > 20 were removed to avoid the impact of collinearity. Further, to provide unbiased estimates of the VPA, microeukaryotic data were Hellinger transformed prior to the analyses [60]. Forward selection procedure was used to select environmental and spatial factors [61]. Finally, VPA was performed using the *varpart* function of the package “vegan” with R (http://www.r-project.org) [20]. It is worth mentioning that although VPA is widely used as a tool to distinguish between niche-based and neutral-based processes for community assembly, several studies based on simulation models revealed that VPA failed to correctly infer the deterministic and stochastic processes on community variation [34, 35, 62]. Therefore, it is difficult to determine the ecological processes totally based on VPA [63], and we employed the Mantel test and neutral community model together with VPA to assess the relative importance of deterministic and stochastic processes in this study.

### Neutral community model for microeukaryotes

To determine the potential importance of stochastic processes on community assembly, we used a neutral community model (NCM) to predict the relationship between OTU detection frequency and their relative abundance across the wider metacommunity [7]. The model used here is an adaptation of the neutral theory [6] adjusted to large microbial populations. In general, the model predicts that taxa that are abundant in the metacommunity will be widespread, since they are more likely to disperse by chance among different sampling sites, whereas rare taxa are more likely to be lost in different sites due to ecological drift (i.e., the stochastic loss and replacement of individuals). In this model, *Nm* is an estimate of dispersal between communities. The parameter *Nm* determines the correlation between occurrence frequency and regional relative abundance, with *N* describing the metacommunity size and *m* being the immigration rate. The parameter *R*^2^ represents the overall fit to the neutral model [7]. Calculation of 95% confidence intervals around all fitting statistics was done by bootstrapping with 1000 bootstrap replicates.

In this study, we separately used the datasets from wet, dry, and both seasons combined. The OTUs from each dataset were subsequently separated into three partitions depending on whether they occurred more frequently than (above partition), less frequently than (below partition), or within (neutral partition) the 95% confidence interval of the NCM predictions. To analyze deviations from the NCM predictions, we compared the composition, diversity, and calculated estimated migration rate (*m*) of neutral and non-neutral (above and below) partitions of microeukaryotes. All computations were performed in R (version 3.2.3) [64].

## Supplementary information

**Additional file 1: Figure S1.** Rarefaction curves of similarity-based operational taxonomic units (OTUs) at 97% sequence similarity level. **Figure S2.** Comparison of microeukaryotic community between wet and dry seasons at supergroup and OTU levels. **Figure S3.** Distance-decay patterns based on Bray-Curtis similarity of microeukaryotic community composition and direct site-to-site (Euclidian) distance in wet and dry seasons, respectively. **Figure S4.** Distance-decay patterns based on Bray-Curtis similarity of microeukaryotic community composition and spatial distance at different taxonomic resolutions in wet and dry seasons, respectively. **Figure S5.** Fit of the neutral community model (NCM) for different microeukaryotic supergroups at OTU level (97% similarity) in wet and dry seasons, respectively. **Figure S6.** Neutral community model fit of microeukaryotes evaluated from genus to kingdom taxonomic resolutions in wet, dry and both seasons, respectively. **Figure S7.** Definition of abundant and rare taxa of microeukaryotic metacommunity in this study. **Figure S8.** The two different types of spatial distances employed in the river microeukaryotic metacommunity study.

**Additional file 2: Table S1.** Summary of environmental factors in the Tingjiang River across wet and dry seasons. **Table S2.** Comparison of environmental factors between wet and dry seasons. **Table S3.** Diversity, predicted richness and Good’s coverage of microeukaryotic community in the 60 samples. **Table S4.** The contribution of each taxa category to the microeukaryotic community in the 60 samples at 97% similarity level. **Table S5.** Mantel tests for the correlations between environmental distance, individual environmental factors and microeukaryotic community compositions in Tingjiang River during wet and dry seasons using Spearman’s coefficients. **Table S6.** Variation partitioning analysis based on partial RDA illustrating the effects of environmental and directional spatial (AEM) factors on the microeukaryotic communities in Tingjiang River. **Table S7.** Variation partitioning analysis based on partial RDA illustrating the effects of environmental and spatial (PCNM) factors on the microeukaryotic communities in Tingjiang River. **Table S8.** Variation partitioning analysis based on partial Mantel test illustrating the effects of environmental and spatial (PCNM) factors on the microeukaryotic communities in Tingjiang River. **Table S9.** The distinct estimated migration rate of neutral and non-neutral partitions of the metacommunity predicted by Sloan neutral model in different seasons. **Table S10.** Mantel tests for the correlations between geographical distance, environmental distance, individual environmental factors and different microeukaryotic community partitions predicted by the neutral community model in Tingjiang River during wet and dry seasons using Spearman’s coefficients. **Table S11.** The distinct estimated migration rate of wet, dry and both seasons’ microeukaryotic supergroups predicted by Sloan neutral model.

## Data Availability

All raw sequences from this study have been submitted to the NCBI Sequence Read Archive (SRA) database under the BioProject number PRJNA414954 and the accession number SRP122256.
