# Correction to: Stochastic processes shape microeukaryotic community assembly in a subtropical river across wet and dry seasons

**DOI:** 10.1186/s40168-019-0763-x

**Published:** 2019-11-14

**Authors:** Weidong Chen, Kexin Ren, Alain Isabwe, Huihuang Chen, Min Liu, Jun Yang

**Affiliations:** 1 Aquatic EcoHealth Group, Key Laboratory of Urban Environment and Health, Institute of Urban Environment, Chinese Academy of Sciences, Xiamen, 361021 China; 2 Fujian Key Laboratory of Watershed Ecology, Institute of Urban Environment, Chinese Academy of Sciences, Xiamen, 361021 China; 3 State Key Laboratory of Marine Environmental Science, Marine Biodiversity and Global Change Research Center, College of Ocean and Earth Sciences, Xiamen University, Xiamen, 361102 China

**Correction to: Microbiome**

**https://doi.org/10.1186/s40168-019-0749-8**

Following publication of the original article [1], the authors reported an error in Fig. 1 and a text on page 13. In Fig. 1, some areas are missing and the correct figure is presented here. On page 13, the sentence should be updated to the following (change has been indicated in bold italics).

**Fig. 1:**
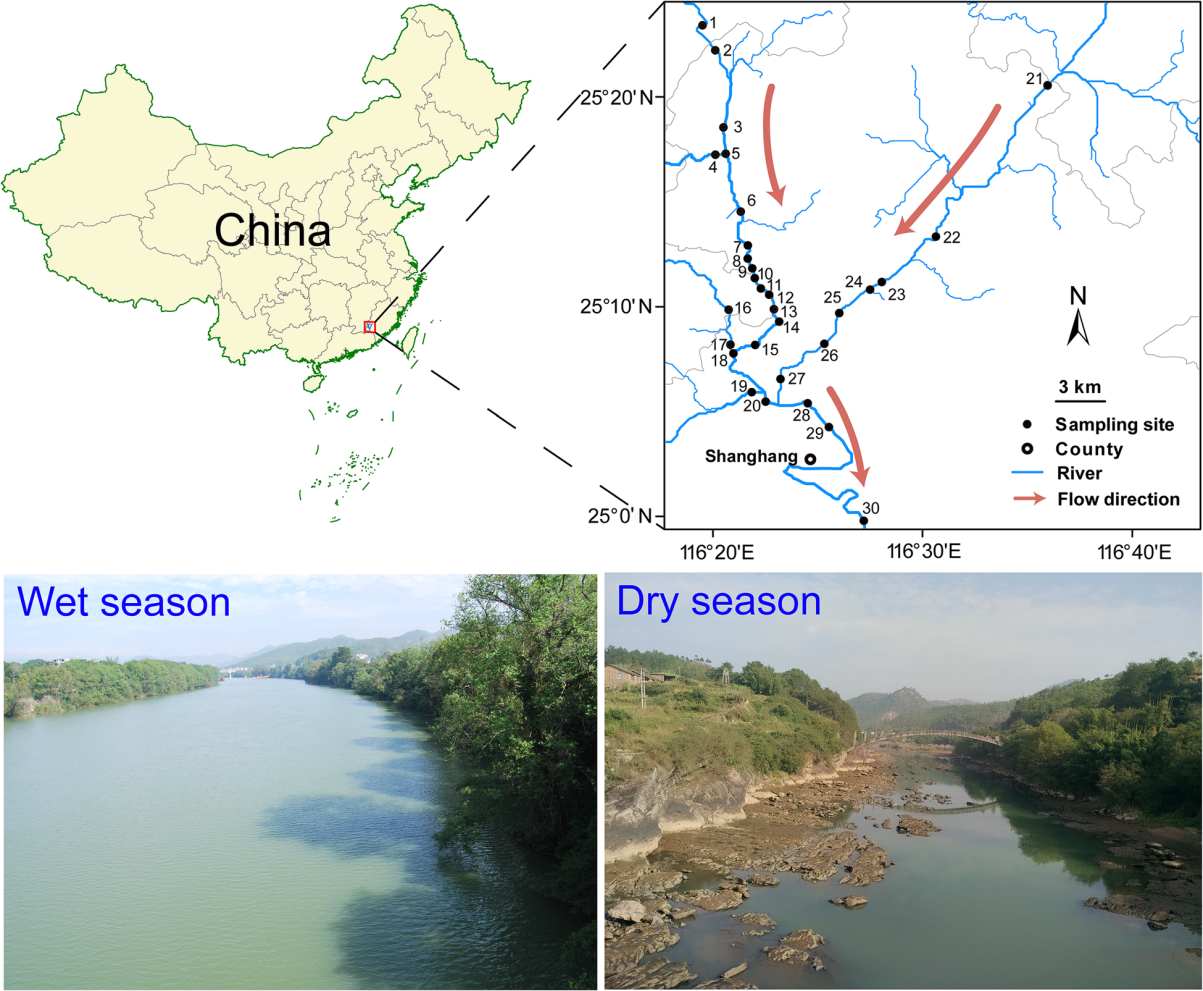
Sketch map of Tingjiang River showing the sampling sites in wet and dry seasons. A total of 60 surface water samples were collected in July and November 2015. The map of Tingjiang River sampling sites was performed using ArcGIS 10.1 (ESRI, Redlands, CA, USA)

From: “These were (1) the dendritic network length (km), which is a measure of the cumulative length of the branching river network ***upstream*** of ***the*** sampling site, and (2) the Euclidian distance (km) based on the longitude and latitude coordinates of each sampling site, which is simply the straight line distance between sampling points. All geographical measures were calculated using ArcGIS (ESRI, Redlands, CA, USA).”

To: “These were (1) the dendritic network length (km), which is a measure of the cumulative length of the branching river network ***(watercourse)*** of ***two*** sampling sites, and (2) the Euclidian distance (km) based on the longitude and latitude coordinates of each sampling site, which is simply the straight line distance between sampling points. All geographical measures were calculated using ArcGIS (ESRI, Redlands, CA, USA).”

The publishers apologise for this error. The original article [1] has been updated.
