# Supplementary material for: Stochastic processes shape microeukaryotic community assembly in a subtropical river across wet and dry seasons
Source: Microbiome. 2019 Oct 22;7:138. doi: 10.1186/s40168-019-0749-8 (PMC6806580; doi:10.1186/s40168-019-0749-8)
Supplement: Supplementary file 1 — Additional file 1: Figure S1. Rarefaction curves of similarity-based operational taxonomic units (OTUs) at 97% sequence similarity level. Figure S2. Comparison of microeukaryotic community between wet and dry seasons at supergroup and OTU levels. Figure S3. Distance-decay patterns based on Bray-Curtis similarity of microeukaryotic community composition and direct site-to-site (Euclidian) distance in wet and dry seasons, respectively. Figure S4. Distance-decay patterns based on Bray-Curtis similarity of microeukaryotic community composition and spatial distance at different taxonomic resolutions in wet and dry seasons, respectively. Figure S5. Fit of the neutral community model (NCM) for different microeukaryotic supergroups at OTU level (97% similarity) in wet and dry seasons, respectively. Figure S6. Neutral community model fit of microeukaryotes evaluated from genus to kingdom taxonomic resolutions in wet, dry and both seasons, respectively. Figure S7. Definition of abundant and rare taxa of microeukaryotic metacommunity in this study. Figure S8. The two different types of spatial distances employed in the river microeukaryotic metacommunity study. [file 40168_2019_749_MOESM1_ESM.pdf]

Journal: Microbiome

Additional file 1 of the article:

Stochastic processes shape microeukaryotic community assembly in a subtropical river across wet and dry seasons

Weidong Chen[1,2,3], Kexin Ren[1,2], Alain Isabwe[1,2], Huihuang Chen[1,2], Min Liu[1,2] and Jun Yang[1,2*]

[1] *Aquatic EcoHealth Group, Key Laboratory of Urban Environment and Health, Institute of Urban Environment, Chinese Academy of Sciences, Xiamen 361021, China*

[2] *Fujian Key Laboratory of Watershed Ecology, Institute of Urban Environment, Chinese Academy of Sciences, Xiamen 361021, China*

[3] *State Key Laboratory of Marine Environmental Science, Marine Biodiversity and Global Change Research Center, College of Ocean and Earth Sciences, Xiamen University, Xiamen 361102, China*

Running title: Neutral assembly of river microeukaryotes

Keywords: Biogeography / Plankton / Microeukaryotic community / Subtropical river / Environmental factors / Neutral community model / Community assembly

*** Correspondence:**
E-mail: jyang@iue.ac.cn (Jun Yang)

This additional information contains:

- 9 Pages
- 8 Figures

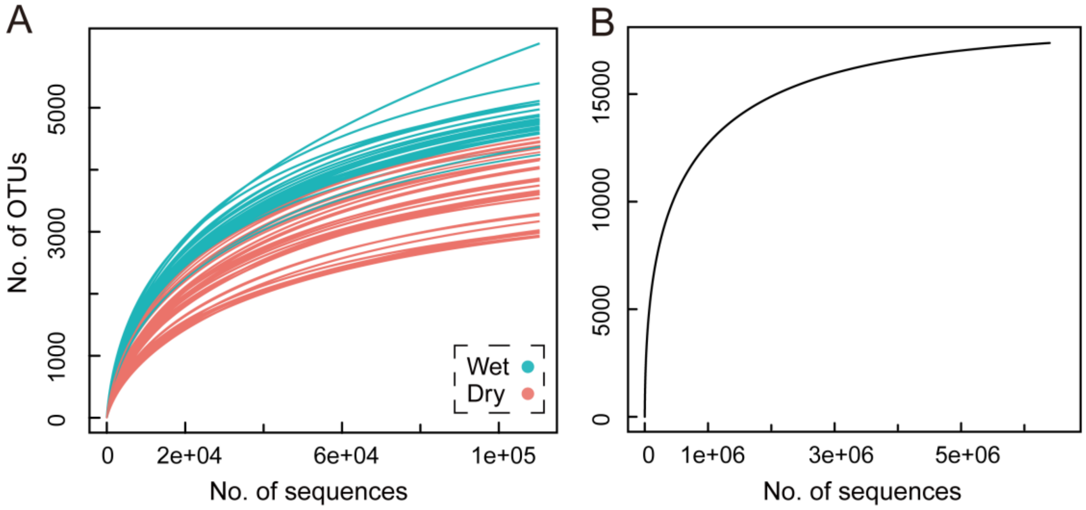

Fig. S1 Rarefaction curves of similarity-based operational taxonomic units (OTUs) at 97% sequence similarity level. (**A**) The individual samples; (**B**) the combined set of 60 samples.

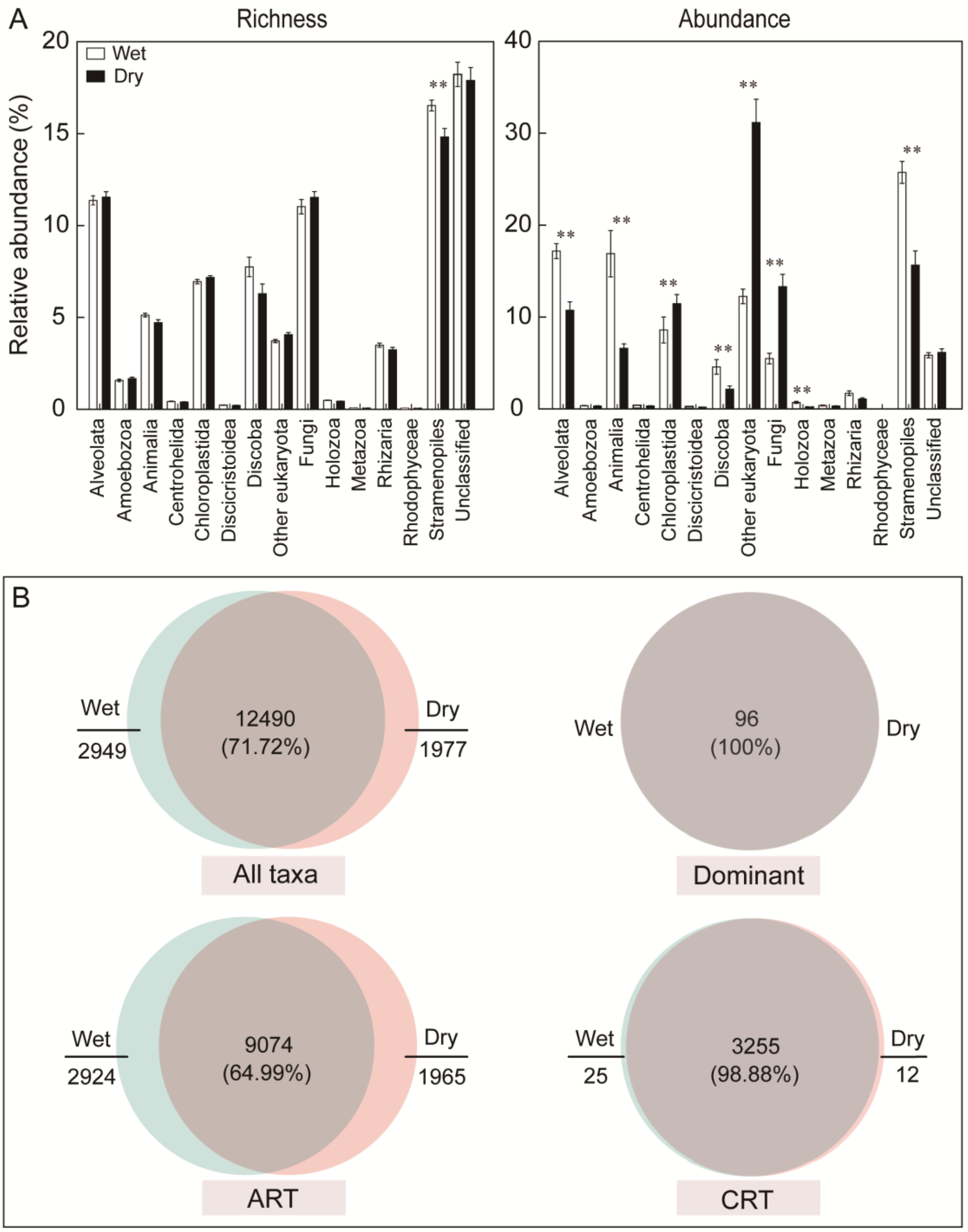

Fig. S2 Comparison of microeukaryotic community between wet and dry seasons at supergroup and OTU levels. (**A**) Relative abundance of richness (OTU numbers) and abundance (sequence numbers) of wet season microeukuayotic taxa compared with dry season microenkuayotic taxa. The other eukaryota represents incertae sedis eukaryota. Unclassified eukaryotes (sequence similarity ≥ 80%). Values are means ± s.e. (n = 30); **, $P < 0.01$ (Student's *t* test). (**B**) Venn diagrams showing the number and percentage of OTUs that are unique and shared between the microeukaryotic taxa between wet and dry seasons. Dominant represents taxa including AAT, CAT and CRAT. AAT: always abundant taxa; CAT: conditionally abundant taxa; CRAT: conditionally rare and abundant taxa; ART: always rare taxa; CRT: conditionally rare taxa.

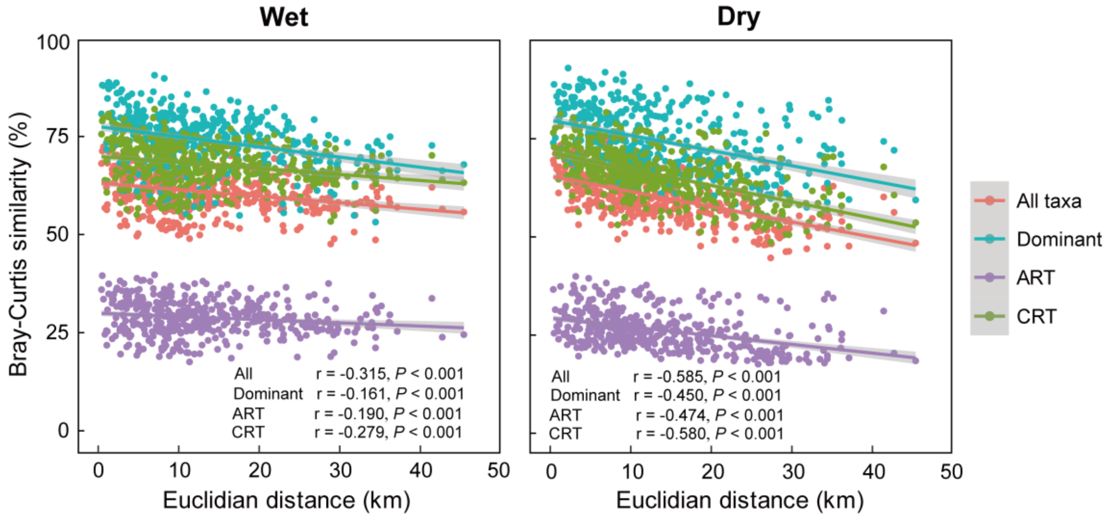

Fig. S3 Distance-decay patterns based on Bray-Curtis similarity of microeukaryotic community composition and direct site-to-site (Euclidian) distance in wet and dry seasons, respectively (n = 435). Dominant represents taxa including AAT, CAT and CRAT. AAT: always abundant taxa; CAT: conditionally abundant taxa; CRAT: conditionally rare and abundant taxa; ART: always rare taxa; CRT: conditionally rare taxa.

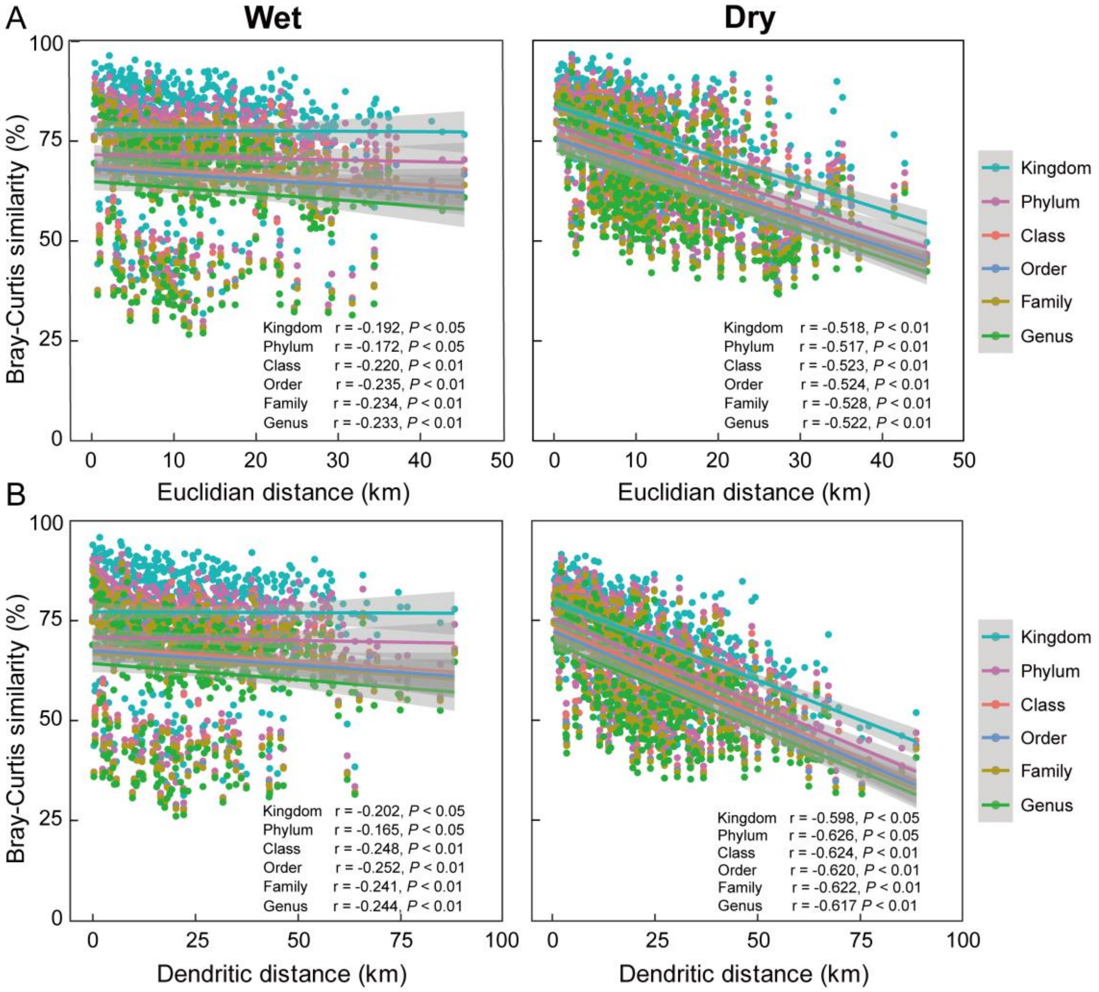

Fig. S4 Distance-decay patterns based on Bray-Curtis similarity of microeukaryotic community composition and spatial distance at different taxonomic resolutions in wet and dry seasons, respectively (n = 435). The spatial distance analyses are conducted based on two distinct methods: (**A**) direct site-to-site (Euclidian) distance; (**B**) cumulative dendritic distance. Taxonomy-based compositional variation is calculated based on the database annotation from genus to kingdom resolutions.

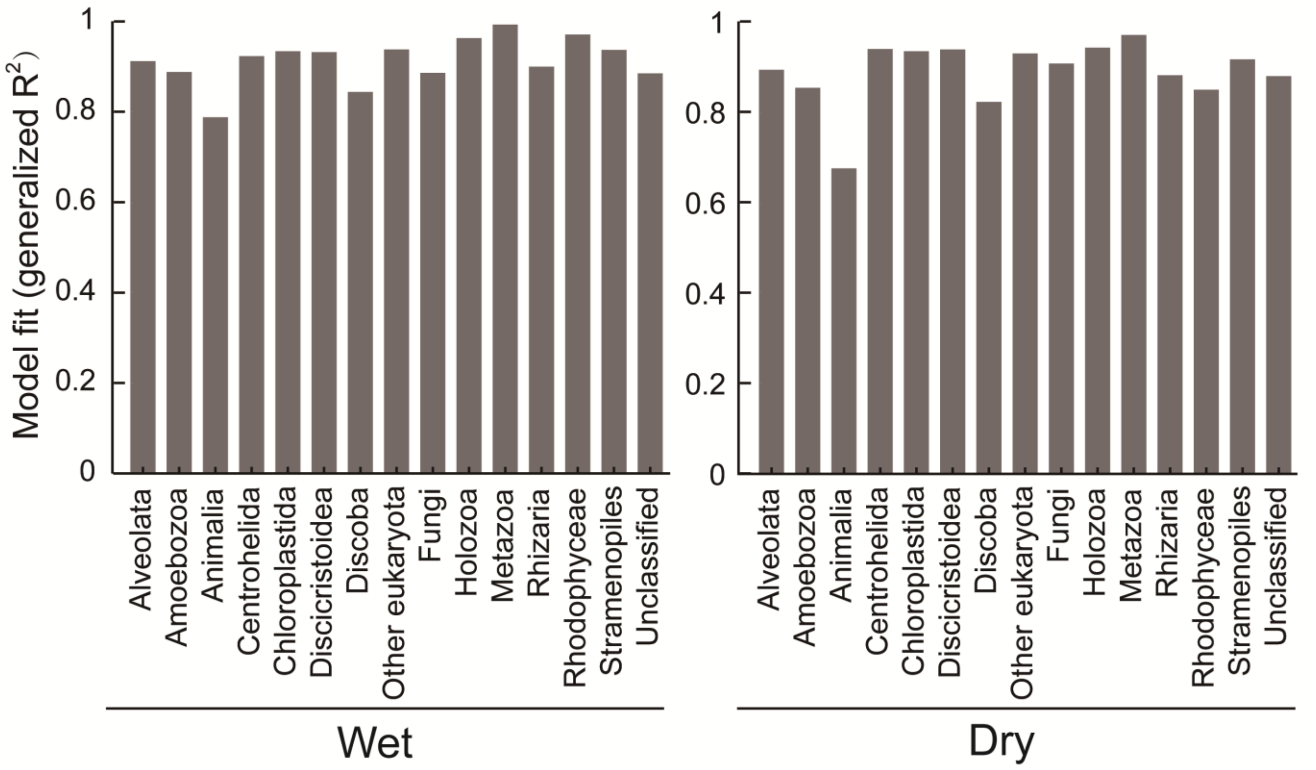

Fig. S5 Fit of the neutral community model (NCM) for different microeukaryotic supergroups at OTU level (97% similarity) in wet and dry seasons, respectively. The other eukaryota represents incertae sedis eukaryota.

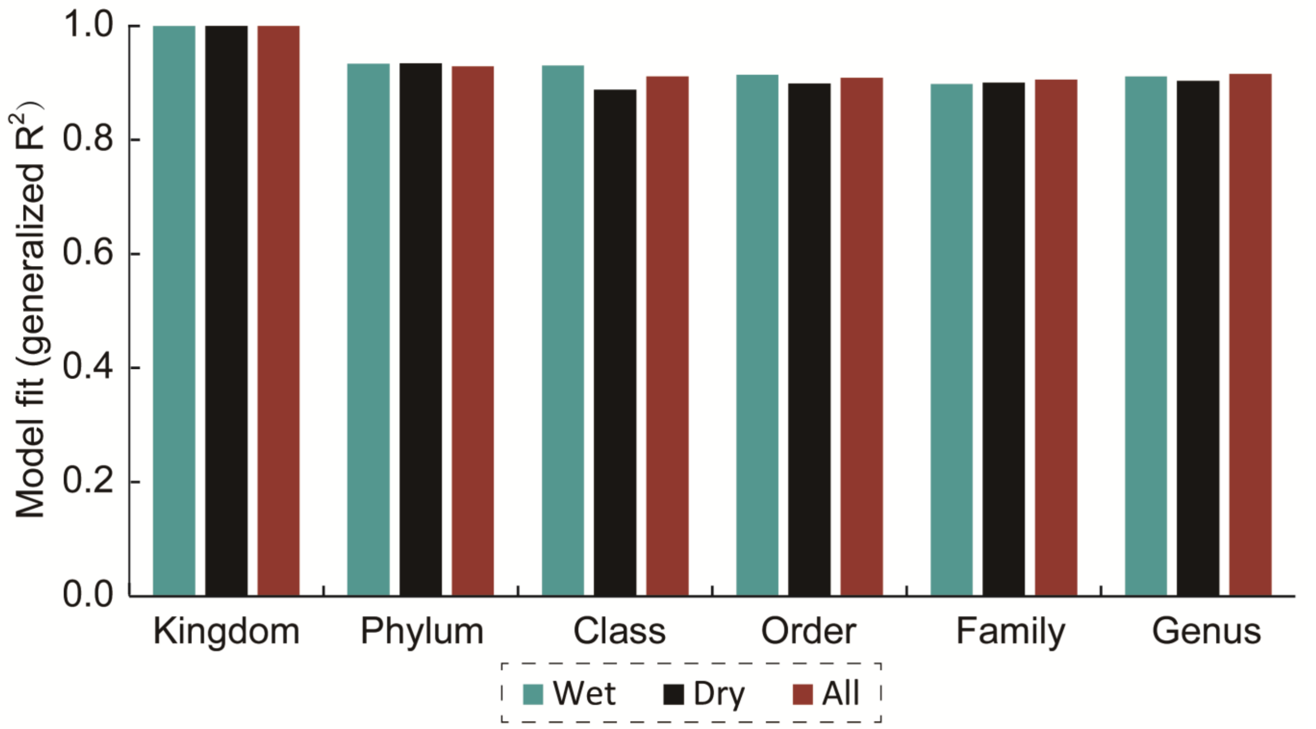

Fig. S6 Neutral community model fit of microeukaryotes evaluated from genus to kingdom taxonomic resolutions in wet, dry and both seasons, respectively. All: both wet and dry seasons.

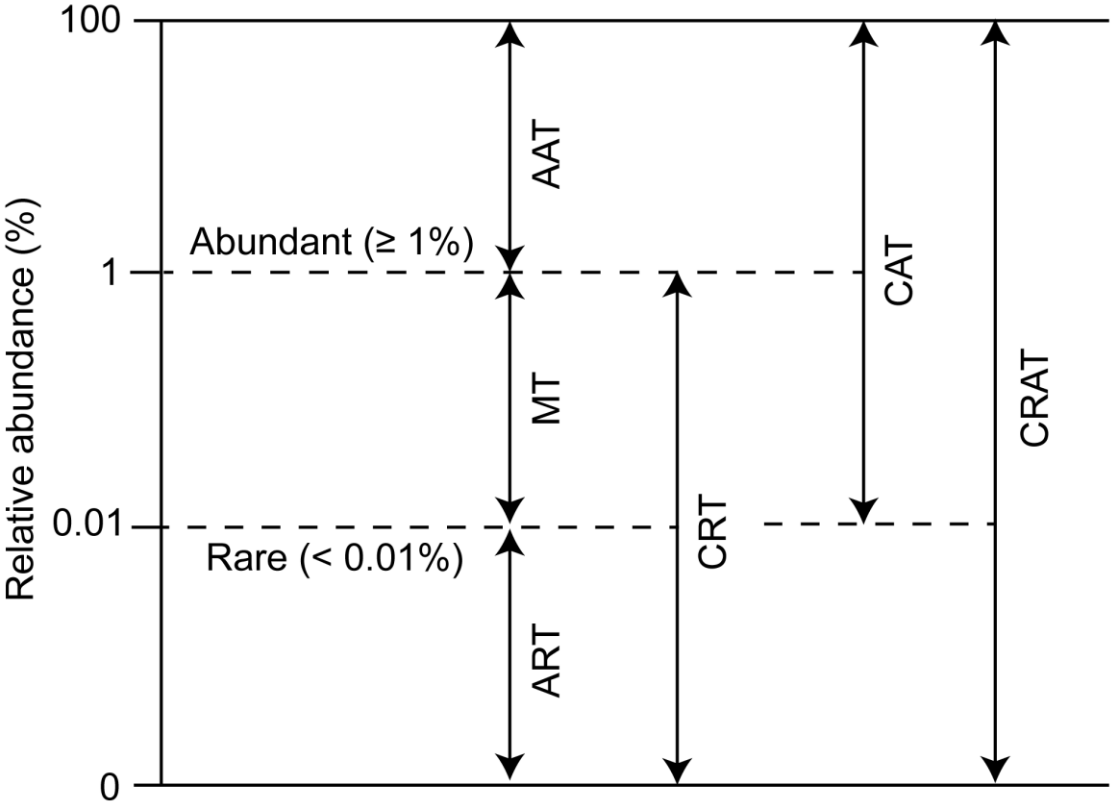

Fig. S7 Definition of abundant and rare taxa of microeukaryotic metacommunity in this study. Always abundant taxa (AAT) were defined as the OTUs with a relative abundance always ≥ 1% in all samples. Conditionally abundant taxa (CAT) were defined as the OTUs with a relative abundance greater than 0.01% in all samples and ≥ 1% in some samples but never rare (< 0.01%). Conditionally rare and abundant taxa (CRAT) were defined as the OTUs with a relative abundance varying from rare (< 0.01%) to abundant (≥ 1%). Moderate taxa (MT) were defined as the OTUs with relative abundance between 0.01% and 1% in all samples. Conditionally rare taxa (CRT) were defined as the OTUs with a relative abundance < 0.01% in some samples but never ≥ 1% in all samples. Always rare taxa (ART) were defined as the OTUs with a relative abundance always < 0.01% in all samples.

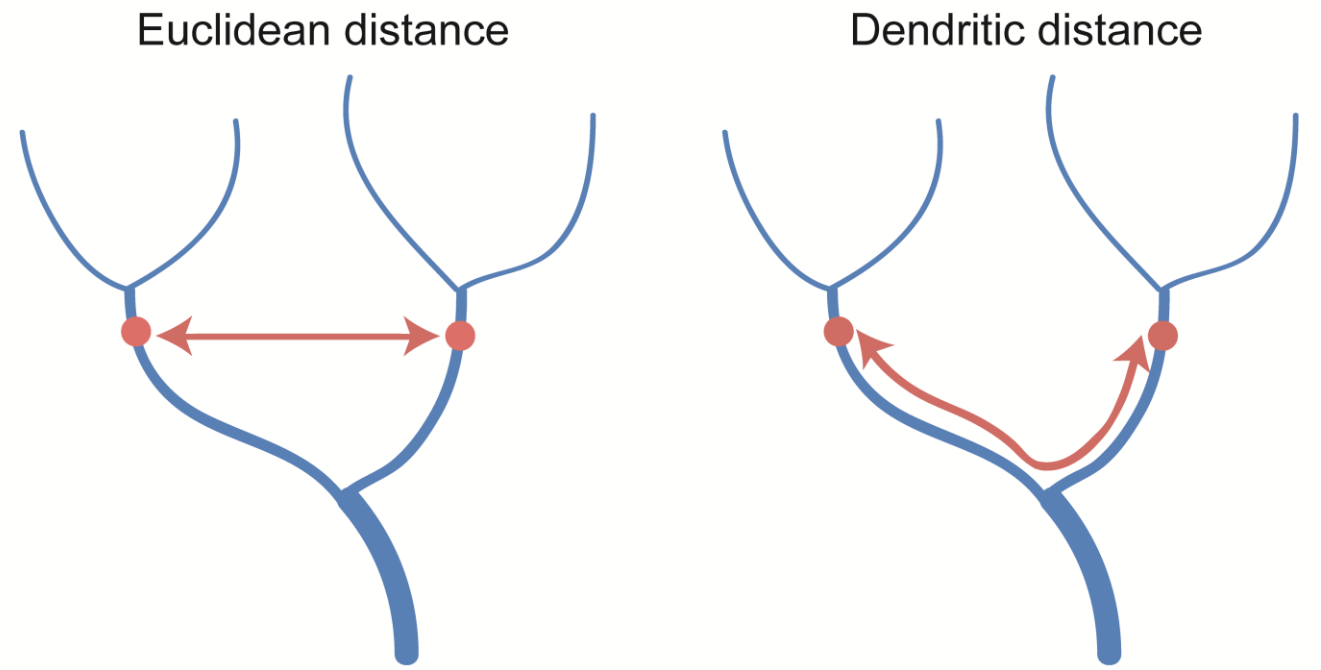

Fig. S8 The two different types of spatial distances employed in the river microeukaryotic metacommunity study. The Euclidian distance represents the straight line distance between two sampling sites; the dendritic distance is a measure of the watercourse (cumulative length of the river network) of two sampling sites.
